# Supplementary material for: Disruption of ultrasonic vocalizations with systemic administration of the non-competitive N-methyl-D-aspartate receptor antagonist MK-801 in adult male mice
Source: Int J Neuropsychopharmacol. 2026 Sep 17;29(10):pyag048. doi: 10.1093/ijnp/pyag048 (PMC13647287; doi:10.1093/ijnp/pyag048)
Supplement: Supplementary file 1 [file supplementary_material_pyag048.zip › Tamura2026_InJNeuropsychopharmacol_Highlights_pyag048.docx]

**Highlights**

- Systemic MK-801 dose-dependently reduced ultrasonic vocalizations recorded from male–female mouse dyads without significantly altering the treated male’s proximity to the female.
- The highest MK-801 dose reduced social approach toward a same-sex conspecific, although preference for the social stimulus over the non-social object remained significant.
- NMDA receptor blockade produced dissociable effects on dyadic vocal activity, social approach, locomotor activity, and excretory outcomes.
